# Products of Compartmental Models in Epidemiology

**DOI:** 10.1155/2017/8613878

**Published:** 2017-08-16

**Authors:** Lee Worden, Travis C. Porco

**Affiliations:** ^1^Francis I. Proctor Foundation, University of California San Francisco, San Francisco, CA, USA; ^2^Department of Ophthalmology, University of California, San Francisco, CA, USA; ^3^Department of Epidemiology and Biostatistics, University of California San Francisco, San Francisco, CA, USA

## Abstract

We show that many structured epidemic models may be described using a straightforward product structure in this paper. Such products, derived from products of directed graphs, may represent useful refinements including geographic and demographic structure, age structure, gender, risk groups, or immunity status. Extension to multistrain dynamics, that is, pathogen heterogeneity, is also shown to be feasible in this framework. Systematic use of such products may aid in model development and exploration, can yield insight, and could form the basis of a systematic approach to numerical structural sensitivity analysis.

## 1. Introduction

Simple epidemic models aim at insight through simplicity; complex models aim at realism through detail [1]. Both simple and complex models are still being developed (e.g., [2–7]). Addition of epidemiological refinements, such as age structure, gender, geographic separation, or pathogen strains, in general changes the behavior of simple models, and thus we must systematically compare models with different features.

In this paper, we show that many structured epidemic models may be described using a straightforward product structure. Such products therefore provide a compact representation for a family of related models and could facilitate model comparison and structural sensitivity analysis. Examples include modeling host susceptibility groups, gender, age structure, multiple subtypes, and geographic separation. Our attention will be restricted to compartmental models [8–10], focusing on mathematical epidemiology [10–17].

The product we describe is related to standard graph products. The relation between compartmental models and graph theoretic or network concepts has been long appreciated [18, 19], and, moreover, Markov processes arising on product spaces have been analyzed by probabilists [20]. The graph structure arises when dynamical variables will be represented as vertices of a graph, representing the number of individuals in a given compartment. Individuals may change state, such changes being represented by an arc from one vertex to another, labeled with the instantaneous rate at which such a transition would occur.

## 2. Motivating Example: Community-Structured Epidemic Model

Consider a simple SI (susceptible to infective) model describing an epidemic with no recovery. Individuals transition from susceptible to infective and never return to the uninfected state. The number of infected individuals is denoted *I* of susceptible individuals *S*.

**Figure 1:**
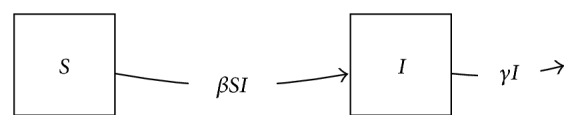
Directed graph diagram of simple *SI* model.

This compartmental model is diagrammed in Figure 1. The corresponding ODE system may be written

(1) dSdt=−βSI,dIdt=βSI−γI.

Here, *β* is a transmission coefficient, and *γ* is the per capita mortality or removal rate due to disease. In this model, we ignore population birth and death due to other causes.

**Figure 2:**
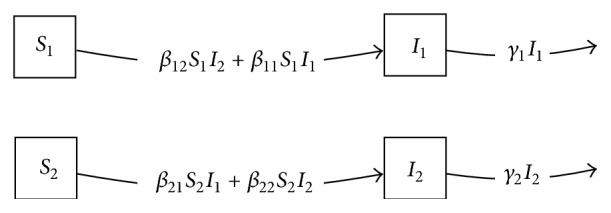
Directed graph diagram of Watson model [22] defined by adding community structure to the *SI* model.

A simple extension to include heterogeneous epidemic dynamics in multiple communities was introduced by Watson et al. [21, 22]. In this model, no migration between communities is assumed. However, individuals in one community cause infection in other communities, with the structure seen in Figure 2. The equations are

(2) dSidt=−∑jβijSiIj,i=1,…,n,dIidt=∑jβijSiIj−γiIi,i=1,…,n,

where *n* is the number of communities modeled. In the Watson model, in general the transmission coefficients may differ when considering transmission to susceptibles in one community from infectives in any community (whether the same or not). Each community is additionally assumed to have a different rate *γ*_*i*_ of removal of infectives due to mortality (or other causes), though these can be assumed to be identical if desired.

This model extends the one-community SI model, by structuring the population into multiple communities. In the following section, we will show that the structured model developed by Watson can be straightforwardly defined as the product of the single-community SI model and a model describing community structure. We will then illustrate other uses of this product, including age structure, gender, heterogeneity of risk, and cotransmission of multiple diseases.

## 3. Graph Products

A directed graph is defined as a set of vertices, each identified by a unique label, together with a set of arrows, or arcs, each connecting a source vertex to a target vertex. In this paper, we are concerned only with directed graphs, not undirected ones. A number of different products of directed graphs are defined, two of which are relevant.

### 3.1. Cartesian Product

Consider finite directed graphs *A* and *B*, with *n*_*A*_ and *n*_*B*_ vertices, respectively. The Cartesian product of these graphs [23] is a graph *A* □ *B* whose vertex set is the set of ordered pairs (*v*, *w*) for all vertices *v* of *A* and *w* of *B* (that is, the Cartesian product of the vertex sets of the factor graphs *A* and *B*). The arcs of *A* □ *B* consist of an arc from (*v*, *w*_*s*_) to (*v*, *w*_*t*_) for every *v*, wherever there is an arc from *w*_*s*_ to *w*_*t*_ in the factor graph *B* and an arc from (*v*_*s*_, *w*) to (*v*_*t*_, *w*) for every *w* wherever there is an arc from *v*_*s*_ to *v*_*t*_ in *A*.

We can speak of “levels” in the sense that each vertex of a factor model corresponds to a subset, or level, of vertices of the product model. The product replicates all the arcs of *B* at every level of *A* and all the arcs of *A* at every level of *B*. Suppose we have two vertices (*a*_*i*_, *b*_*j*_) and (*a*_*k*_, *b*_*j*_), whose second coordinate is the same, that is, which map to the same level of *B*; it will be helpful to call these “siblings” and to say they “descend” from a common “factor vertex” *B*, similarly for vertices with the same first coordinate.

**Figure 3:**
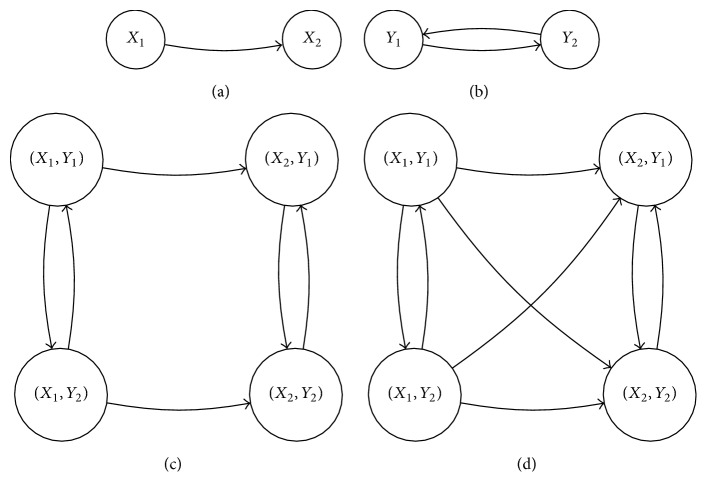
(a, b) Example directed graphs *A* and *B*, respectively; (c) Cartesian product *A* □ *B*; (d) strong product *A*⊠*B*.

Figures 3(a), 3(b), and 3(c) illustrate two directed graphs and their Cartesian product, respectively.

More generally, graphs with multiple arcs joining a pair of vertices can be defined, and the Cartesian product definition above can be applied in this case as well.

### 3.2. Strong Product

The strong product of two directed graphs *A* and *B* includes more arcs than the Cartesian product [23]. This product *A*⊠*B* has the same vertex set, the Cartesian product of the factors' vertex sets, but in addition to the arcs of the Cartesian product graphs, it also includes all arcs from (*v*_*s*_, *w*_*s*_) to (*v*_*t*_, *w*_*t*_) where there is an arc from *v*_*s*_ to *v*_*t*_* and* an arc from *w*_*s*_ to *w*_*t*_.

Figure 3(d) illustrates the strong product of the graphs of Figures 3(a) and 3(b).

The symbols □ and ⊠ for these operations are chosen to evoke the structure of the product graphs, as illustrated in Figures 3(c) and 3(d). These graph products are discussed in more detail in the Appendix.

## 4. Products of Models

### 4.1. Linear Compartmental Models

A compartmental model (whether in population biology, epidemiology, or pharmacology) is often represented by a diagram such as in Figure 1, which has the form of a directed graph (formally, a directed multigraph) with labels on arcs. Multiple arcs may connect a single pair of compartments, representing multiple processes influencing that transition with potentially different rates. As before, the vertices of the graph are compartments and its arcs are transitions, with labels specifying the transition rates. (We consider a compartmental model to be an abstract object isomorphic to its directed multigraph diagram.) As is well known, a compartmental model diagram can be represented by a system of ordinary differential equations or a continuous Markov jump process (among others). (For instance, a compartmental model with a single compartment *N*, with a single inflow with rate Λ and outflow with rate *μN*, can be represented by the simple stochastic immigration-death process [24] or by the elementary ordinary differential equation model *dN*/*dt* = Λ − *μN*.)

The class of linear compartmental models we consider in this section includes the ordinary differential equation models of the form

(3) $$\begin{array}{c} \frac{dX}{dt}=a+MX, \end{array}$$

where *X* is a vector of *n* state variables, *a* is a vector of constant inflows, and *M* is an *n* × *n* transition rate matrix. The general compartmental model, with sources and sink terms, can be represented in the same graphical way by considering special source and sink vertices in the graph.

A Cartesian product of linear compartmental models will be defined in a way that is similar to the Cartesian product of graphs. Suppose that *A*_1_, *A*_2_,…, *A*_*K*_ are the states in model *A*; let *B*_1_,…, *B*_*L*_ be the states of model *B*. The Cartesian product of *A* with *B* will have states (*A*_*i*_, *B*_*j*_) with *i* = 1,…, *K* and *j* = 1,…, *L*. Two states (*A*_*i*_, *B*_*j*_) and (*A*_*i*_, *B*_*j*′_) are* siblings* in the same* level A*_*i*_ of the product. If we began, for example, with an epidemic model with states susceptible, infective, removed (SIR), and wished to construct a product with a geographic model of multiple regions, we would expect to have susceptibles, infectives, and removed individuals in each region.

The Cartesian product of graphs, as we saw, replicates each arc of each factor graph for each vertex of the other factor graphs. In a compartmental model of a population system, this would correspond to the very common assumption of competing independent exponential risks. For example, consider once again a simple SIR epidemic model, with infection and recovery, and a model of two communities with migration between them. In a Cartesian product of the two, we may wish to allow infection and recovery within each community as well as migration of susceptibles from one community to another, migration of infectives, and migration of recovered individuals. In the product model, infectives in one community, for example, should be able to move to the other community or recover within their own community—a feature exactly reflected in the structure of a Cartesian graph product.

However, note that, in general, we may well wish to assume differences in these parameters. We may wish to assume, for example, that recovery rates are higher in one community or that migration rates of infectives are lower than for susceptibles. Unlike a Cartesian graph product, a Cartesian product of compartmental models must take into account the arc labels, which are the transition rates; in general, new parameters are necessarily introduced.

We propose the following definition for a Cartesian product of linear compartmental models. If a transition in model *B* from *B*_*j*_ to *B*_*j*′_ occurs with rate *γ*, then for every state *i* in model *A*, a transition in the product model occurs from (*A*_*i*_, *B*_*j*_) to (*A*_*i*_, *B*_*j*′_) at rate *γ*_*i*_. Similarly, if a transition in model *A* from *A*_*i*_ to *A*_*i*′_ occurs with rate *θ*, then for every state *j* in model *B*, a transition in the product model occurs from (*A*_*i*_, *B*_*j*_) to (*A*_*i*′_, *B*_*j*_) at rate *θ*_*j*_.

The presence of sources and sinks does not add any fundamental complications. If a transition in model *B* from *B*_*j*_ to a sink occurs with rate *μ*, then for every state *i* in model *A*, a transition from (*A*_*i*_, *B*_*j*_) to the sink occurs with rate *μ*_*i*_ (similarly,* mutatis mutandis*, for transitions in *A* to a sink). Finally, if a transition from a source to state *B*_*j*_ in model *B* occurs at rate Λ, then in the product model, for every state *i* in model *A*, a transition from a source to (*A*_*i*_, *B*_*j*_) occurs at rate Λ_*i*_ (and similarly for transitions from the source which appear in model *A*). These, and only these, transitions constitute the product model.

See the Appendix for more detail on the Cartesian product of linear models.

## 5. Epidemic Models

How can products like the Cartesian and strong products of graphs be used in formulating epidemic models? As we shall see, the product reviewed above can be extended to this case as well. We must extend the Cartesian product of compartmental models to allow interaction between different populations. (We note that similar considerations apply in the more general ecological modeling setting, including Lotka-Volterra predator-prey and competition equations, but we will not pursue these applications.)

### 5.1. Structured SI Model

In this section, we return to the classical Watson epidemic model, representing the SI epidemic in multiple regions. We will extend the Cartesian product of linear compartmental models, showing that the Watson model is a product of the simple SI model and a geographic model. In this special case, the geographic model will have no transitions at all.

The SI model of the transmission process is the one discussed above, with two states *S* and *I*, and transitions as pictured in Figure 1.

**Figure 4:**
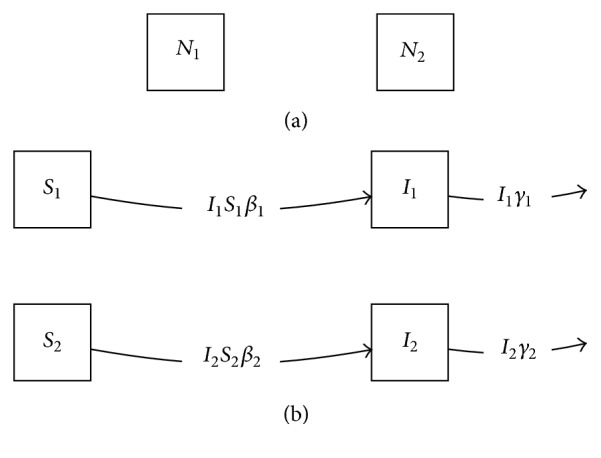
(a) Factor model representing community structure with no migration; (b) simple Cartesian product formula applied to the *SI* and community structure models, giving an incorrect result.

We now define a factor model which distinguishes individuals by community, to be combined with the SI model. If there are *n* communities, let *N*_1_, *N*_2_,…, *N*_*n*_ be the number of individuals in each community. If no migration takes place, and we ignore demographic turnover, this model corresponds to the differential equation system *dN*_*k*_/*dt* = 0 for all *k*. For simplicity, we will illustrate only the *n* = 2 case (Figure 4(a)).

The state space of the product model will consist of the numbers of susceptibles and infectives in community 1 and community 2, ordered pairs such as (*S*, *N*_1_), which can be given names *S*_1_, *I*_1_, *S*_2_, and *I*_2_. Naive application of the Cartesian product for compartmental models would begin with the observation that the SI factor model includes a transition from *S* to *I* at rate *βI*. We would then iterate over the levels *j* = 1,2 of the community model. We need a transition from (*S*, *N*_1_) ≡ *S*_1_ to (*I*, *N*_1_) ≡ *I*_1_, but at what rate? Generalizing the Cartesian product formula given above in the most direct way produces a model with transition rates *β*_1_*S*_1_*I*_1_ and *β*_2_*S*_2_*I*_2_, corresponding to the graph seen in Figure 4(b). This is a technically valid compartmental model, but it does not account for potential transmission between infectives in one community (e.g., *I*_2_) and susceptibles in the other (e.g., *S*_1_).

We must therefore extend the Cartesian product of compartmental models. In this example, we must take into account that the rate of transmission between a susceptible and an infective individual depends on the community membership of the infective as well as that of the susceptible. The extended definition is as follows.

As above, let *A* and *B* be models. The state space for the extended Cartesian product *C* is, as before, the Cartesian product of the state spaces of *A* and of *B*. For *A*, the transition rates include functional forms *f*(*A*_*i*_, *A*_*j*_), that is, functional dependencies on one or more states of *A*.

In the example of the Watson epidemic model, we have the following. The transition rate denoting transmission events in the SI model has rate *f*(*S*, *I*) = *βSI*. We will construct the product model using the rule that, from every compartment *S*_*i*_ ∈ {*S*_1_, *S*_2_}, that is, for every compartment descended from the *S* compartment, there is a transition to its corresponding sibling descended from the *I* compartment, at rate *f*_*ij*_(*S*_*i*_, *I*_*j*_) = *β*_*ij*_*S*_*i*_*I*_*j*_, for every *I*_*j*_ ∈ {*I*_1_, *I*_2_}. Note that the infective compartment *I*_*j*_ in this definition is distinct from the target vertex of the transition—the transition arc points from *S*_*i*_ to *I*_*i*_, on the level *i* of the source vertex, but the infective compartment *I*_*j*_ ranges over levels *j* of the product model independently of the source—and this distinction is crucial to defining the correct set of transitions.

**Figure 5:**
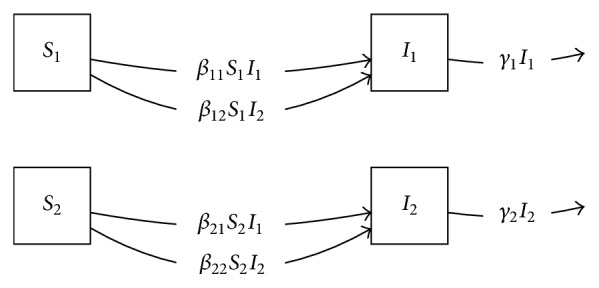
Directed multigraph diagram of Watson model [22] defined by applying the extended Cartesian product operation to the *SI* and two-community models.

Where our earlier definition constructs one arc from each *S* compartment to its corresponding *I* compartment, this definition constructs one arc from each *S* compartment to its *I* compartment* for each* infective compartment that can transmit to those susceptibles. This yields the model shown in Figure 5. This extended Cartesian product yields two arcs for transitions from *S*_1_ to *I*_1_, the first reflecting our intent that individuals in community 1 can cause infections in their own community and the second reflecting transmission to community 1 from community 2. This can be canonically represented as a single arc whose rate is the sum of the rates in the individual arcs, which, in this example, is (*β*_11_*I*_1_ + *β*_12_*I*_2_)*S*_1_, as in the original presentation of this model by Watson [22]. Similarly, two arcs appear for transitions from *S*_2_ to *I*_2_. Thus, the extended Cartesian product correctly represents the Watson model as a product of a within-community epidemic process and a geographic model.

Here we provide a formal definition of this product.

**Definition 1 .**

A simple Cartesian product of two compartmental models *A* and *B* is a compartmental model *A* □ *B* whose set of compartments is the set of ordered pairs (*X*_*i*_, *Y*_*j*_) for every compartment *X*_*i*_ of *A* and *Y*_*j*_ of *B*.

For every arc *α* of model *A*, with per capita transition rate *f*^*α*^(*X*_*s*_, *Z*_1_,…, *Z*_*k*_), source compartment *X*_*s*_, and target compartment *X*_*t*_, the arcs of the product model include all arcs of the form

(4) $$\begin{array}{c} x_{s}\overset{f_{i,\ldots }^{\alpha }\left(x_{s},z_{1},\ldots ,z_{k}\right)}{\to }x_{t}, \end{array}$$

where *x*_*s*_, *z*_1_,…, and *z*_*k*_ range over all compartments of the product model descending from *X*_*s*_, *Z*_1_,…, and *Z*_*k*_, respectively, and *x*_*t*_ is the compartment descending from *X*_*t*_ that is otherwise on the same level as *x*_*s*_, together with the corresponding arcs derived from the arcs of factor model *B*. The subscripts of *f*_*i*,…_^*α*^ distinguish the different arcs by providing the names of the levels to which all of the product compartments *x*_*s*_, *z*_1_,…, *z*_*k*_ belong. The set of arcs of the product model consists of only the above arcs.

The product transition rates *f*_*i*,…_^*α*^(*x*_*s*_, *z*_1_,…, *z*_*k*_) can be defined as needed, to generate an appropriately concise form for the transition rate functions, set unneeded transition rates to zero, or to do other works of specifying the details of the combined epidemic dynamics. Examples below demonstrate several ways of using these functions to construct specific models.

Before introducing a series of examples of product models with epidemiological application, we note that the product compartments, defined as ordered *n*-tuples such as (*S*, *N*_1_), can be assigned variable names such as *S*_1_ in a number of ways. We will use several different naming conventions in our examples. Likewise the parameters such as *β* and *γ* need to be mapped in product transitions to differentiated variables such as *β*_12_ and *γ*_1_, as appropriate to the application. We consider this to be part of the definition of the function *f*_*ij*_^*α*^(*S*_*i*_, *I*_*j*_) and other rate functions.

### 5.2. Community Model Featuring Demographics

We note that the disease process factor model may be generalized to include demographic turnover (“vital dynamics”). For example, this may feature a constant inflow of new susceptibles and an exponential mortality or removal; the SI model could be expressed in the form

(5) dSdt=Λ−βSI−μS,dIdt=βSI−μI.

Here, Λ is a constant recruitment rate, and *μ* is a per capita death rate (see, e.g., [10]). If sources and sinks are considered to be special compartments, the above definition encompasses such inflow and outflow transitions. If we construct the extended Cartesian product model of this SI process with the same community model, we obtain the correct product model, with differential equations

(6) dSidt=Λi−∑jβijSiIj−μiSi,dIidt=∑jβijSiIj−μiIi.

**Figure 6:**
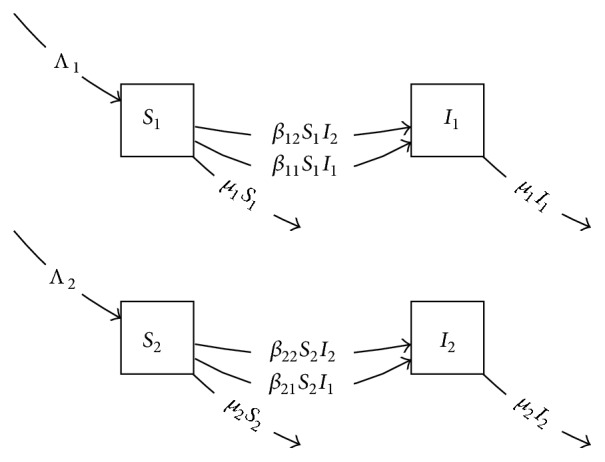
Product model of *SI* process in two communities.

This model is illustrated in Figure 6.

Other elaborations of the epidemic model can be combined with regional models in the same way, including the SIS process (used, for instance, to model gonorrhea (e.g., [25]) and more recently to model infectious trachoma [26]), the SIR model, more complex variants (e.g., [27–29] out of a vast literature), or even models featuring vector-borne transmission (e.g., [30–33]). Useful factor models can include regional models with transportation, host genetics [10, 34, 35], gender, vaccination status, multiple risk groups (e.g., high and low risk of infection), or the presence of a second infectious agent.

### 5.3. Compartmental Aging

Age-structured models are frequently used in analysis of disease transmission to reflect changes in susceptibility, frequency of complications, or mixing patterns which depend on age. Compartmental model product structure can easily reflect these features, as we illustrate in the following example. Consider the standard SIR model to be the first factor model:

(7) dSdt=−βSI,dIdt=βSI−γI,dRdt=γI,

where *β* and *γ* are transmission and recovery rates as above.

We then use the following compartmental aging process as the second factor model:

(8) dA0dt=Λ−αA0−μ0A0,dA1dt=αA0−αA1−μ1A1,dA2dt=αA1−αA2−μ2A2.

Here, *α* is simply the rate of aging (one year per year), Λ is a constant recruitment rate, and constants *μ*_*i*_ are age-class-specific per capita mortality rates.

The number of age compartments could be chosen to be any positive integer, in principle. Numerically, the use of compartmental aging can yield large stiff systems of equations, but compartmental aging approximates the use of McKendrick-von Foerster equations for aging.

**Figure 7:**
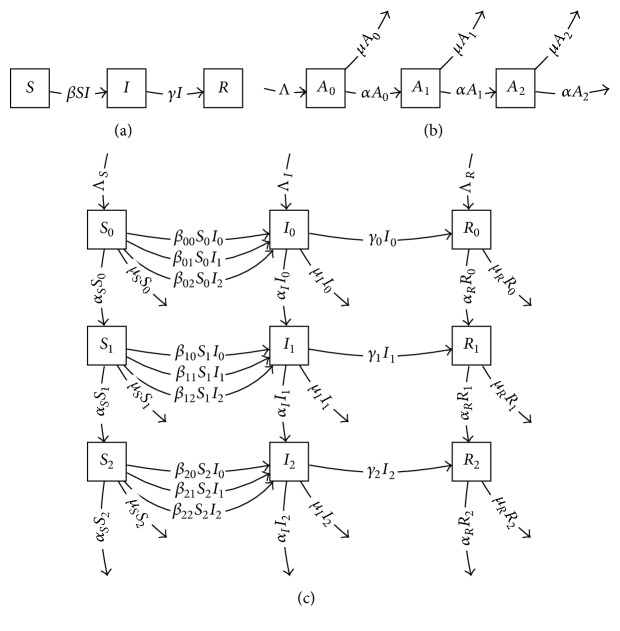
(a) *SIR* model, (b) age structure model, and (c) product of *SIR* model with age structure model.

These two models and their Cartesian product model are shown in Figures 7(a), 7(b), and 7(c). In the product model, inflow term Λ_*I*_ represents vertical transmission, while Λ_*R*_ would represent individuals immune at birth. Either of these rates can be set to zero for specific applications.

We note that, in this example, we have built recruitment and mortality into the aging model, while in the previous section we included them in the transmission model. There is flexibility in where to include these demographic processes, depending on what subscripts one wishes to have attached to their rates in the product model. If needed they can even be included in multiple factor models and assigned to constant values including zero as appropriate in the product.

### 5.4. Risk-Stratified STI Model

Sexual behavior is highly heterogeneous, with some individuals having far more partners per unit time than others. Moreover, such individuals may preferentially mix with similar individuals. The epidemiological role of a relatively small group of highly active people in transmission of a sexually transmitted infection (STI) was explored in a mathematical model of gonorrhea [36], and similar approaches were used in HIV modeling [37].

Consider the following simple example. Suppose that we begin with the factor model

(9) dSdt=Λ−βcpSIN−μS,dIdt=βcpSIN−γI−μI

representing disease transmission in a population of MSM (men who have sex with men) [10]. Here *β* represents the transmission probability per partnership, *c* equals a susceptible individual's rate of acquiring new sexual partners, and *p* is the probability that a susceptible individual's partner is chosen from a specific population of infectives (in the basic factor model, there is only one population *I* of infectives, and in that model this probability *p* is taken to have a constant value of 1, but these probabilities will be nontrivial in the product model, in which there are multiple infective populations). Here *γ* is disease-specific per capita mortality, Λ is a constant inflow rate of susceptibles, and *μ* is the disease-independent mortality rate. We will multiply this model by a second factor model in which the population is divided into a high risk group *A* and a low risk group *B*, with transition rates *ρ* and *σ* between them:

(10) dAdt=−ρA+σB,dBdt=ρA−σB.

**Figure 8:**
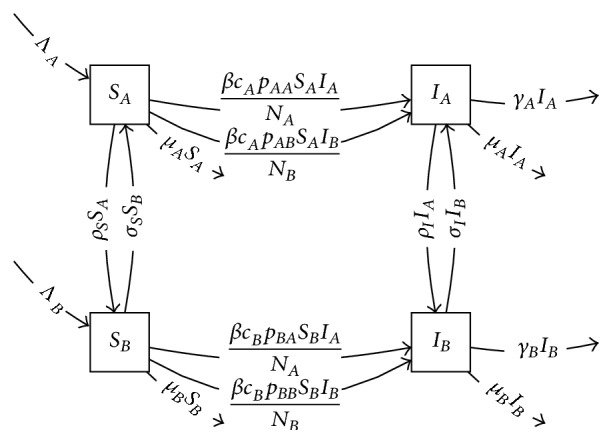
Product of *SI* model with risk-structure model.

The product model is shown in Figure 8. This model can then represent the presence of a high risk core group with a higher rate *c*_*A*_ of acquiring partners than the other, as well as nonrandom mixing between the groups, expressed by the probabilities *p*_*AA*_, *p*_*AB*_, and so forth. Because the mixing probabilities *p*_*AA*_ and *p*_*AB*_ for a susceptible individual in group *A* must sum to one, and likewise *p*_*BA*_ and *p*_*BB*_, we could replace *p*_*AB*_ and *p*_*BB*_ by 1 − *p*_*AA*_ and 1 − *p*_*BA*_, but it is not necessary to do so. As formulated, this system keeps the biologically distinct roles of *c* and *p* separate, although in some circumstances it may be desirable to combine them, while respecting the constraint that (*S*_*A*_ + *I*_*A*_)*c*_*A*_*p*_*AB*_ = (*S*_*B*_ + *I*_*B*_)*c*_*B*_*p*_*BA*_ [38]. However, one may desire to have the quantities *p* as functions of the state variables, reflecting that partner choice probabilities may depend on the dynamically varying group sizes [37, 39], in which case it is advantageous to retain them as separate parameters so that they can be replaced by more complex expressions straightforwardly.

### 5.5. Gender in STI Models

Modeling heterosexual transmission of an STI may proceed by dividing the population into males and females. Such a model can be developed along lines very similar to the risk model in the previous section. We may begin with a similar transmission factor model, here shown as an SIS process:

(11) dSdt=Λ−βcpSIN−μS+γI,dIdt=βcpSIN−γI−μI.

The second factor model will be simply

(12) $$\begin{array}{c} \frac{dF}{dt}=\frac{dM}{dt}=0, \end{array}$$

where we assume no transitions from male to female or vice versa. In constructing the product model, we incorporate the assumption of heterosexual-only transmission by defining the partner choice probabilities *p*_*ij*_ to be one for opposite-gender combinations (*p*_*FM*_, *p*_*MF*_) and zero for the same-gender combinations. The product model is then

**Figure 9:**
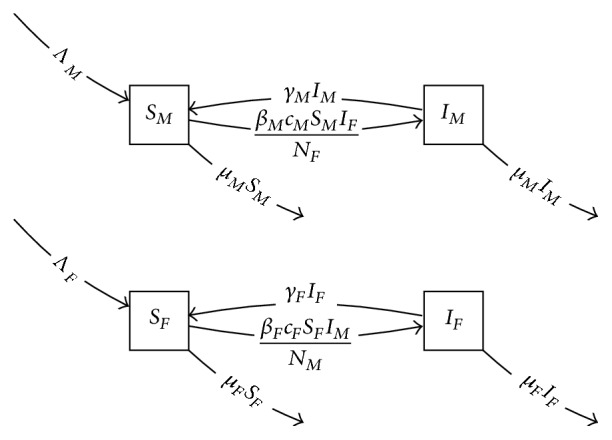
Product of *SI* model with static two-gender model.

(13) dSFdt=ΛF−βFcFIMNMSF−μFSF+γFIF,dSMdt=ΛM−βMcMIFNFSM−μMSM+γMIM,dIFdt=βFcFIMNMSF−μFIF−γFIF,dIMdt=βMcMIFNFSM−μMIM−γMIM,

as seen in Figure 9.

### 5.6. Interacting Transmission of Leprosy and Tuberculosis

**Figure 10:**
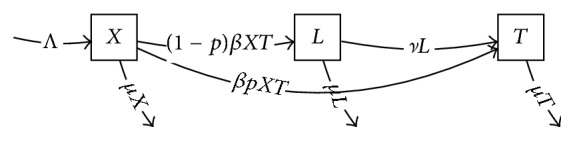
Compartmental model of tuberculosis transmission.

The extended Cartesian product of compartmental models can be applied to problems involving two separate infectious disease processes. In the joint leprosy-tuberculosis model appearing in [40], the epidemiological effects of cross-immunity between two mycobacterial species were analyzed using a compartmental model. This model may be represented as a product of two-factor models, the first being a simple tuberculosis model based on susceptible (*X*), latent TB (*L*), and active tuberculosis (*T*):

(14) dXdt=Λ−μX−βXT,dLdt=1−pβXT−μL−νL,dTdt=pβXT+νL−μT,

where Λ is a recruitment rate, *μ* is an overall mortality rate, *ν* is a rate of progression of latent tuberculosis to active disease, *β* is a transmission coefficient (hazard rate per infective) (*β*_*T*_ in the paper), and *p* is the probability a newly infected individual will develop active tuberculosis rapidly instead of becoming latently infected with tuberculosis (Figure 10).

**Figure 11:**
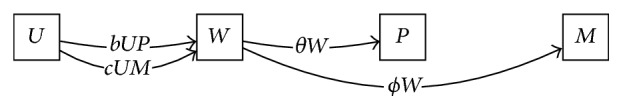
Compartmental model of leprosy transmission.

The second factor model represents the progression of leprosy from susceptible *U*, to latent infection with leprosy (*W*), and to multibacillary disease (*M*) or paucibacillary disease (*P*). The leprosy factor model is then (Figure 11)

(15) dUdt=−bP+cMU,dWdt=bP+cMU−θ+ϕW,dPdt=θW,dMdt=ϕW,

where here *b* is the transmission coefficient for paucibacillary leprosy (*β*_*P*_ in the paper), *c* is the transmission coefficient for lepromatous leprosy (*β*_*M*_ in the paper), *θ* is the rate at which latently infected individuals develop paucibacillary disease (*ν*_*P*_ in the paper), and *ϕ* is the rate at which latently infected individuals develop multibacillary disease (*ν*_*M*_ in the paper).

**Figure 12:**
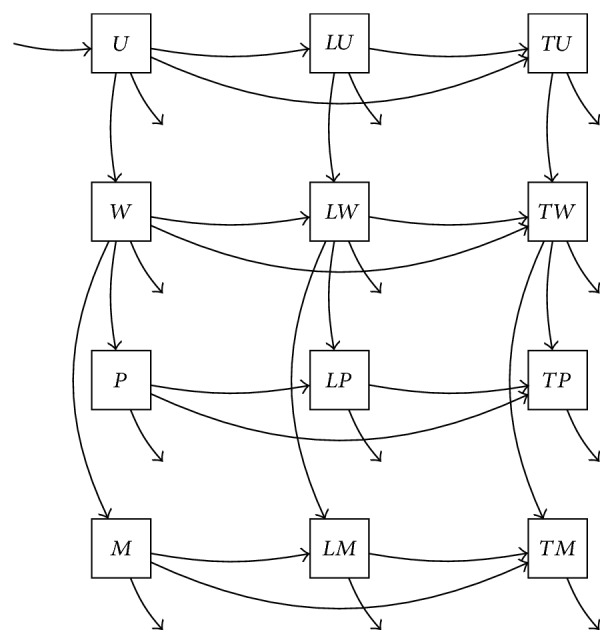
Cartesian product of leprosy (Figure 11) and tuberculosis (Figure 10) models, describing interaction of the two transmission processes. Multiple arrows and labels are suppressed for legibility.

The product model (Figure 12) represents the epidemiological interference of the two closely related mycobacterial infections. Individuals latently infected with one may have partial immunity against the other. This product structure could be applied to other settings such as HIV-TB interactions (e.g., [41]).

## 6. Strong Products

The extended Cartesian product is too restrictive when constructing models of multiple diseases. For instance, one may be infected by two pathogens during a single encounter with a dually infected person. Thus, it may be necessary to allow individuals to proceed to dual infection directly from the susceptible class without passing through the singly infected states. The extended Cartesian product defined earlier does not permit this possibility.

Just as the Cartesian product of graphs can be extended to a strong product of graphs, an analogous strong product is possible for products of compartmental models. As we will show below, a strong product of compartmental models will permit derivation of multistrain or multidisease models featuring simultaneous transmission.

As an example, consider the following simple SIS epidemic model, which we might apply to transmission of* Chlamydia trachomatis*, the etiologic agent of trachoma (a blinding disease) [42, 43]. In principle, multiple strains of the trachoma agent can circulate [44]. Consider a model of a single strain, in which *S* is the number of susceptibles and *I* is the number of infectives:

(16) dSdt=−βSIN+γI,dIdt=βSIN−γI,

where *β* is a transmission coefficient, *γ* is a recovery rate, and *N* = *S* + *I* is the total population.

We can construct a multistrain model with partial cross-immunity [45, 46] using a suitably defined strong product, defined as follows. Let *A* and *B* be models, with states labeled *A*_*i*_ and *B*_*j*_, respectively. The vertices of the strong product model *A*⊠*B* are, as in the previously defined products, the Cartesian product of the vertex sets of the factor models. Every arc of each factor model gives rise to one or more arcs within each level of the product model, as in the other products, one for each interaction with product compartments. There are also additional, diagonal arcs in the product model that cross levels, representing more than one of the factor model's transitions taking place simultaneously.

**Figure 13:**
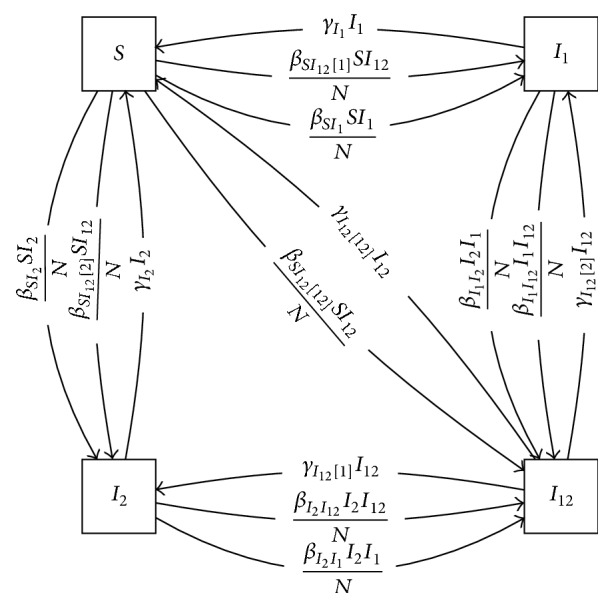
Strong product of two strains' *SIS* dynamics.

In Figure 13, we present the strong product of the above SIS model with itself.

The arcs of the Cartesian product of models are present, representing infection of an individual by either strain 1 or 2, but there are additional arcs as well, including the diagonal transition from *S* to *I*_12_ representing simultaneous transmission of both strains 1 and 2 to a fully susceptible individual in a single encounter with an individual carrying both strains.

Unlike the Cartesian product, here a single interaction between compartments can manifest in multiple transitions. The interaction between examples *S* and *I*_12_ can result in transmission of either or both strains, and these cases are represented by three arcs in the diagram, with transmission rates *β* distinguished by brackets.

A formal definition of the strong product of compartmental models, which generates the above example product, is as follows.

**Definition 2 .**

A strong product of two compartmental models *A* and *B* is a compartmental model *A*⊠*B* whose set of compartments is the set of ordered pairs (*X*_*i*_, *Y*_*j*_) for every compartment *X*_*i*_ of *A* and *Y*_*j*_ of *B*. Models *A* and *B* are considered to be distinct models for the purpose of this definition, and each model's transitions are considered distinct from the other, even when a product is taken of a model with itself.

For every set *α* = {*α*_1_,…, *α*_*p*_} of factor models' transitions, belonging to distinct factor models, each with source compartment *X*_*s*_^*α*_*i*_^, target compartment *X*_*t*_^*α*_*i*_^, and per capita transition rate *f*^*α*_*i*_^(*X*_*s*_^*α*_*i*_^, *Z*_1_^*α*_*i*_^,…, *Z*_*k*_*i*__^*α*_*i*_^), the product model includes all arcs of the form

(17) $$\begin{array}{c} x_{s}\overset{f_{i,\ldots }^{\alpha_{1},\ldots ,\alpha_{p}}\left(x_{s},z_{1},\ldots ,z_{k}\right)}{\to }x_{t}, \end{array}$$

where *x*_*s*_ ranges over the compartments of the product model that descend from all the factor vertices {*X*_*s*_^*α*_1_^,…, *X*_*s*_^*α*_*p*_^}, each *z*_*j*_ ranges over the product compartments that descend from all vertices in the set {*Z*_*j*_^*α*_*i*_^}, and *x*_*t*_ descends from all of {*X*_*t*_^*α*_*i*_^} and is otherwise on all the same levels as *x*_*s*_. The arcs of the product model are only those generated by the above definition. As previously, the subscripts *i*,… to the rate function *f* distinguish the different product arcs by indicating the levels to which all the function's arguments belong.

In our SI example, we have defined the rate functions *f* to produce appropriate products of transmission (*β*) and recovery (*γ*) transitions, with distinct but compact subscripts, and to omit transitions in which transmission of one strain occurs simultaneously with recovery from the other one.

## 7. Exploration of a Family of Models

**Figure 14:**
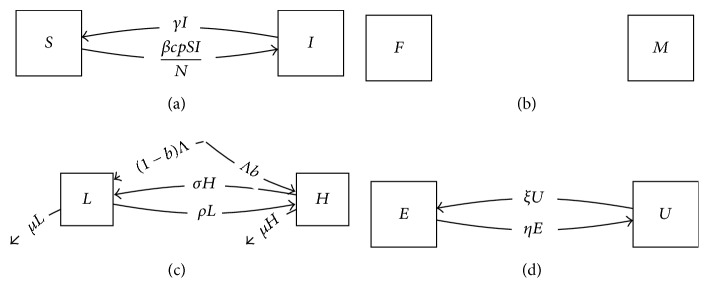
Component models for gonorrhea process: (a) transmission model, a classic SIS process; (b) gender model, a male-female binary system, with the assumptions that nonbinary proportions and transition rates are low; (c) risk model, consisting of high and low risk groups; (d) exposure model, consisting of groups unexposed and exposed to screening.

In this section, we illustrate the use of the extended Cartesian product in model development and exploration, using a model of targeted screening for gonorrhea as a simple example. Such a model can be expressed using four components: a natural history model, a partitioning of the population by gender, a division into low and high risk groups, and a process of screening of individuals (Figure 14). For the natural history model, we will use the simple SIS process as in [25] for illustration, while recognizing that for some STIs a more complex transmission model may be needed, for example, to reflect partial immunity [47]. Because within- and between-gender transmission can vary greatly, we include a division of the model population by gender, with the assumption that rates of gender transition and proportions of nonbinary individuals are small in comparison to the model dynamics. We include a high and low risk group as in [36], with transitions between the risk groups, and finally we include an exposure model tracking the individuals exposed to a control measure such as frequent screening [48].

**Figure 15:**
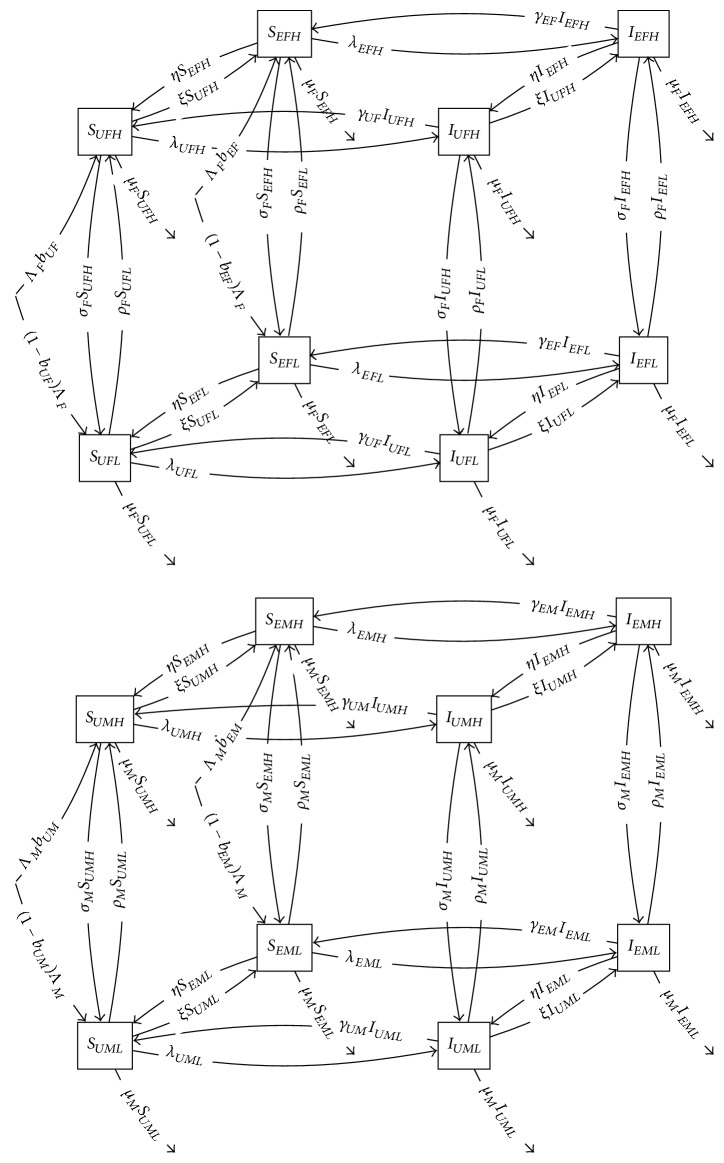
Four-level product model of gonorrhea transmission with stratification into gender, risk, and exposure categories. Transmission rate is abbreviated here for readability: *λ*_*abc*_ = *S*_*abc*_*c*_*bc*_∑_*def*_*β*_*be*_*p*_*bcef*_*I*_*def*_, where *a*, *b*, *c* and likewise *d*, *e*, *f* range over exposure, gender, and risk groups, respectively.

The product of these four models (constructed by extending the above definition of the extended Cartesian product of two models or by taking a product of products) has sixteen compartments and describes a process of transmission with rates affected by the genders, risk group membership, and exposure status of both susceptibles and infectives (Figure 15). The product structure naturally generates a process that includes both homosexual and heterosexual transmission. As drawn here, the effect of the screening program is expressed by changes in the removal rate *γ* such that screened individuals are removed from the infective state more quickly than those who are not screened.

**Figure 16:**
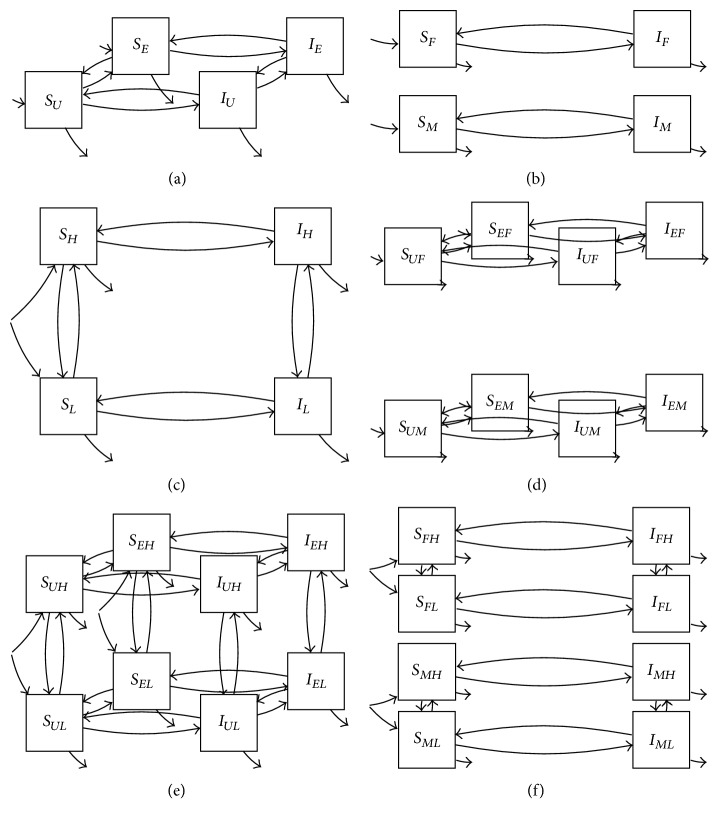
Some candidate models for explanation of recorded transmission dynamics: (a) transmission with exposure only; (b) with gender only; (c) with risk groups only; (d) with exposure and gender; (e) with exposure and risk groups; and (f) with gender and risk groups.

Using the extended Cartesian product definition of this model, it is straightforward to generate partial products using subsets of the set of four factor models shown in Figure 14, yielding a spectrum of models of intermediate complexity (Figure 16), which can be evaluated on their ability to fit observed data. Methods to evaluate the goodness of fit of such a model to data might include least squares (e.g., [49]) or likelihood methods (e.g., [50]).

**Figure 17:**
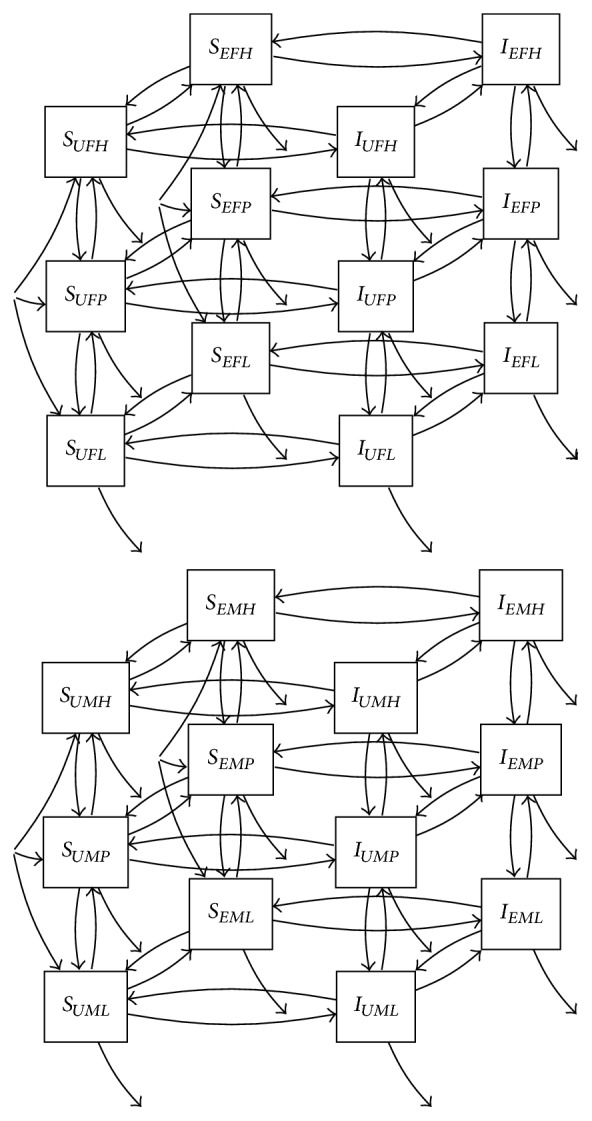
Transmission dynamics with three risk groups (low, partial, and high), together with gender and exposure categories.

More importantly, it is also straightforward using this formulation to generate models with greater detail, for example, by using more than two risk groups (Figure 17). In this way, models with arbitrarily large numbers of risk groups can be straightforwardly and systematically evaluated for goodness of fit to find the best description of the true process available in this framework, a process which cannot be undertaken without an automated model generation framework of this sort.

## 8. Discussion

Products of compartmental models, defined as straightforward generalizations of graph products, represent useful operations in developing epidemiological models. Similar mathematical structures arise from addition of age structure, gender, geographic differences, or other forms of heterogeneity to an epidemic model. Such similarities reveal the presence of a “design pattern” [51] that is captured by the extended Cartesian products we define here.

The products presented in this paper by no means represent the full range of possible products of models. For the products we presented, the state space of the product model is the Cartesian product of the state spaces of the factor models. Some cases may require only a subset of this: consider an HIV model in which the infective classes are structured by CD4 count and viral load classes, which are not relevant to the susceptible classes. Such examples can be easily handled by straightforward generalizations of the products given in this paper. More complex products are required when the state space of the product model must include history (for example, the order in which individuals were infected by pathogen strains).

The extended Cartesian product is well suited to the operation of adding host heterogeneity to an epidemic model, and so it may facilitate automated generation of a family of epidemic models. Similarly, the extended strong product is suited to the process of adding pathogen heterogeneity to an epidemic model. We have developed software to implement these products. All models and figures in this paper were generated by this software, which is freely available as a module for the Sage mathematics computing system [52]. This software enables systematic numerical exploration of a large family of related models, to automate evaluation of specific refinements of an epidemic process for relevance to observed dynamics, and could form the basis of a systematic approach to numerical structural sensitivity analysis.

## Figures and Tables

**Figure 18:**
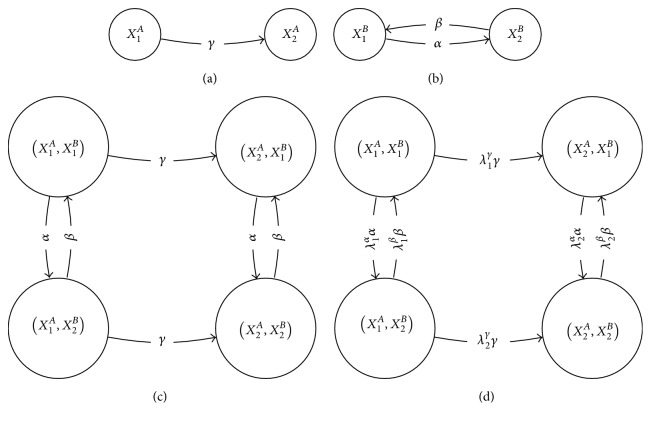
(a) Diagram of states and transition rates, for example, Markov model; (b) diagram for the second example, Markov model; (c) diagram of states and transition rates for simple Cartesian product of models; (d) diagram of states and transition rates for general Cartesian product of models, as defined in the text.

## Appendix

### A. Graph Products

Formally a directed graph is a set of vertices together with arcs, defined as a set of ordered pairs of vertices. We consider graph products for which the vertices of the graph product are formed from the Cartesian product of the vertices of each of the factor graphs. Notationally, if *S*_1_, *S*_2_,…, *S*_*n*_ are sets of vertices, then their Cartesian product is the set *S*_1_ × *S*_2_ × ⋯×*S*_*n*_ = {(*s*_1_, *s*_2_,…, *s*_*n*_)∣*s*_1_ ∈ *S*_1_, *s*_2_ ∈ *S*_2_,…, *s*_*n*_ ∈ *S*_*n*_}, with the elements of *S*_1_ × *S*_2_ × ⋯×*S*_*n*_ being tuples of elements of the component sets *S*_1_, *S*_2_,…, *S*_*n*_.

For application to mathematical models, the arrows (arcs) will represent transition rates between states represented by vertices. Each arc will therefore require an associated label. We allow multiple arcs between the same vertices and thus we must use multigraphs, represented as a set {(*v*, *w*, *e*)}⊆*V* × *V* × *E*. Here, *V* is the vertex set of the graph and *E* is its set of arc labels. Each of these tuples is visualized as an arrow from *v* to *w* with label *e* (which we may denote as $\left\{v\overset{e}{\to }w\right\}$).

The Cartesian product *G*_1_ □ *G*_2_ □⋯□ *G*_*n*_ of directed graphs *G*_1_, *G*_2_,…, *G*_*n*_ is a graph whose vertex set is the Cartesian product *V*_1_ × *V*_2_ × ⋯×*V*_*n*_ of the vertex sets *V*_*i*_ of each graph *G*_*i*_ and whose arcs are of the form $\left(v_{1},v_{2},\ldots ,v_{i},\ldots ,v_{n})\overset{e}{\to }(v_{1},v_{2},\ldots ,w_{i},\ldots ,v_{n}\right)$, where the two tuples are identical in all but the *i*′th position and where there is an arc connecting *v*_*i*_ to *w*_*i*_ in *G*_*i*_. In this paper, the products defined will yield the arc labels (transition rates) in the product graph.

For each vertex in one of the factor models, each vertex in the other model is replicated. If the first factor is a graph with vertices *A* and *B*, with an arc from *A* to *B*, and the second is a graph with vertices 1 and 2 and an arc from 1 to 2, then the product graph contains vertices which could be denoted as *A*1, *A*2, *B*1, and *B*2. Each arc of one graph is replicated for every vertex of the other. Thus, for example, the arc from 1 to 2 in the second factor graph corresponds to an arc from *A*1 to *A*2 for the first vertex of the first model and also to an arc from *B*1 to *B*2 for the second vertex. Similarly, the arc from *A* to *B* in the first model corresponds to an arc from *A*1 to *B*1 (for arc 1 of the second model) and from *A*2 to *B*2 (for the other).

The strong product (or strong Cartesian product) of graphs *G*_1_,…, *G*_*n*_, written *G*_1_⊠*G*_2_⊠⋯⊠*G*_*n*_, is the graph whose vertex set is the Cartesian product of the graphs' vertex sets, which has an arc from (*v*_1_,…, *v*_*n*_) to (*w*_1_,…, *w*_*n*_) if and only if, for every *i*, either there is an arc from *v*_*i*_ to *w*_*i*_, or *v*_*i*_ = *w*_*i*_. The Cartesian product *G*_1_ □ *G*_2_ □⋯□ *G*_*n*_ of directed graphs *G*_1_, *G*_2_,…, *G*_*n*_ is a graph whose vertex set is the Cartesian product *V*_1_ × *V*_2_ × ⋯×*V*_*n*_ of the vertex sets *V*_*i*_ of each graph *G*_*i*_ and whose arcs are of the form $\left(v_{1},v_{2},\ldots ,v_{i},\ldots ,v_{n})\overset{e}{\to }(v_{1},v_{2},\ldots ,w_{i},\ldots ,v_{n}\right)$, where the two tuples are identical in all but the *i*′th position and where there is an arc connecting *v*_*i*_ to *w*_*i*_ in *G*_*i*_.

#### A.1. Adjacency Matrices

The Cartesian product graph can also be defined by its adjacency matrix. Let the adjacency matrices of finite graphs *A* and *B*, respectively, be *M*_*A*_ and *M*_*B*_. Let *I*_*A*_ and *I*_*B*_ be identity matrices of the same size as *M*_A_ and *M*_*B*_, respectively. The Cartesian product graph *A* □ *B* has adjacency matrix *M*_*C*_ = *M*_*A*_ ⊕ *M*_*B*_ = *M*_*A*_ ⊗ *I*_*B*_ + *I*_*A*_ ⊗ *M*_*B*_, where ⊗ is the Kronecker product. Writing *M*_*C*_′ = *M*_*B*_ ⊕ *M*_*A*_ yields an adjacency matrix for the product graph which is the same, except for the ordering of the vertices in the product (and the order in which the Cartesian product of sets of vertices is taken). More generally, graphs with multiple arcs between two vertices can be defined, in which case the elements *a*_*ji*_ of the adjacency matrix record the number of directed arcs to *j* from *i*. The Cartesian product definition above can be applied in this case as well.

The strong product of two directed graphs *A* and *B* includes more arcs than the Cartesian product [23]. This product *A*⊠*B* has the same vertex set, the Cartesian product of the factors' vertex sets, but in addition to the arcs of the Cartesian product graphs, it also includes all arcs from (*v*_*s*_, *w*_*s*_) to (*v*_*t*_, *w*_*t*_), where there is an arc from *v*_*s*_ to *v*_*t*_* and* an arc from *w*_*s*_ to *w*_*t*_. Using the same notation as above, the adjacency matrix of the strong product graph is *M*_*D*_ = *I*_*A*_ ⊗ *M*_*B*_ + *M*_*A*_ ⊗ *I*_*B*_ + *M*_*A*_ ⊗ *M*_*B*_.

Products of more than two graphs can be represented by analogous, though more tedious, matrix operations.

#### A.2. Linear Compartmental Models

Suppose we consider a compartmental model with states *X*_0_, *X*_1_,…, *X*_*k*_ represented by the first-order linear system

(A.1) $$\begin{array}{c} \dot{X}=a+MX, \end{array}$$

where *a* is a vector of exogenous inflow terms and *M* is a transition rate matrix. In general, *M* could contain sink terms; for example, let *X*_1_ represent the number of individuals in a population with constant recruitment Λ and constant per capita mortality, so that ${\dot{X}}_{1}=\Lambda -\mu X_{1}$. In this case, *M* is a 1 × 1 matrix [−*μ*].

Consider two continuous time chains, with state spaces *X*_*i*_^*A*^, *i* = 1,…, *n*_*A*_ and *X*_*j*_^*B*^, *j* = 1,…, *n*_*B*_, respectively. Suppose that the transition rate matrix for each is given by *M*_*A*_ and *M*_*B*_, respectively (assumed time-independent, for simplicity). A transition rate matrix may contain both positive (inflow) terms and negative (outflow) terms. A transition to *X*_*k*_^*A*^ from state *X*_*i*_^*A*^ at rate *λ* will be represented in *M*_*A*_ by a term in the *i*, *i*th element of *M*_*A*_ of −*λ* and a term in the *k*, *i*th element of *M*_*A*_ of *λ*

(A.2) $$\begin{array}{c} M_{A}=\left[\begin{array}{ccc} -\lambda & \mu & \sigma \\ \lambda & -\mu -\theta & 0\\ 0 & \theta & -\sigma \end{array}\right]. \end{array}$$

Any transition rate matrix can be represented as a sum over arcs:

(A.3) MA=−λ00λ00000+0μ00−μ0000+0000−θ00θ0+00σ00000−σ.

A Cartesian product of two Markov chains *A* and *B* can then be defined as follows. We will denote the arcs of model *A* by *α*_*A*_; for each arc there is a matrix *M*_*A*_^*α*_*A*_^ whose elements are 0, except for the inflow and outflow represented by that arc, as illustrated above. The decomposition of the transition rate matrix *M*_*A*_ by arcs is then *M*_*A*_ = ∑_*α*_*A*__*M*_*A*_^*α*_*A*_^. Similarly, the transition rate matrix *M*_*B*_ of model *B*, decomposed by arcs, is *M*_*B*_ = ∑_*α*_*B*__*M*_*B*_^*α*_*B*_^. If *I*_*A*_ and *I*_*B*_ are identity matrices of the same dimension as *M*_*A*_ and *M*_*B*_, respectively, a special case of the Cartesian product can be written

(A.4) $$\begin{array}{c} M_{C}=\underset{\alpha_{A}}{\sum }I_{B}⊗M_{A}^{\alpha_{A}}+\underset{\alpha_{B}}{\sum }M_{B}^{\alpha_{B}}⊗I_{A}. \end{array}$$

Figure 18 depicts two such models (Figures 18(a) and 18(b)) and their product as defined here (Figure 18(c)). This special case only represents a model in which the two chains behave completely independently.

To obtain a more general product, we replace the identity matrices with general diagonal matrices. The elements of the diagonal matrix are not assumed identical. We let Λ_*A*_^*α*_*B*_^ be such a diagonal matrix of the same dimension as *M*_*A*_, representing a scaling matrix for each arc of model *B* for every state of model *A*. Similarly, Λ_*B*_^*α*_*A*_^, a matrix of the same dimension as *M*_*B*_, is a scaling matrix for each arc of model *A* for every state of model *B*. A more general product is then expressed by

(A.5) $$\begin{array}{c} M_{C}=\underset{\alpha_{A}}{\sum }\Lambda_{B}^{\alpha_{A}}⊗M_{A}^{\alpha_{A}}+\underset{\alpha_{B}}{\sum }M_{B}^{\alpha_{B}}⊗\Lambda_{A}^{\alpha_{B}}, \end{array}$$

producing a product process like the one pictured in Figure 18(d).

Note that a given arc connecting two vertices may, in applications, represent two separate processes. For instance, we may have a compartment representing live individuals and another dead and wish to model the rate of death due to two causes, say *μ*_1_ and *μ*_2_. Canonically, we may represent the total transition from live to dead as a single arc with rate *μ*_1_ + *μ*_2_ (assuming independent competing risks), but in the decomposition above, if we represent the transition by two separate arcs, the Cartesian product formula may be applied in the same way.

Let us use the notation *z* ∝ *Z* to indicate that compartment *z* descends from factor compartment *Z*. The transition matrix for the extended Cartesian product model is

(A.6) MC=∑α∑z1∝Z1α⋯∑zk∝ZkαΛBα,z1,…,zk⊗MAαz1,…,zk+∑α∑z1∝Z1α⋯∑zk∝ZkαMBαz1,…,zk⊗ΛAα,z1,…,zk,

where *k* is considered to depend on *α*.
